# Surviving Colonies of *Pseudomonas aeruginosa* Isolated In Vivo from Infected, Antibiotic-Treated *Galleria mellonella* Larvae Acquire an Antibiotic-Tolerant Phenotype

**DOI:** 10.3390/antibiotics14050507

**Published:** 2025-05-15

**Authors:** Alex McCormack, Joanne K. Hobbs, Paul R. Johnston, Peter J. Coote

**Affiliations:** 1 Biomedical Sciences Research Complex, School of Biology, University of St Andrews, The North Haugh, St Andrews KY16 9ST, Fife, UK; am572@st-andrews.ac.uk (A.M.); jkh26@st-andrews.ac.uk (J.K.H.); 2 School of Medicine, University of St Andrews, The North Haugh, St Andrews KY16 9TF, Fife, UK; prj3@st-andrews.ac.uk

**Keywords:** antibiotic tolerance, persister(s), *Galleria mellonella*, whole genome sequencing, recurring infection

## Abstract

**Background:** The aim of this work was to induce the formation of antibiotic-tolerant and/or persister cells in vivo using antibiotic therapy on *Galleria mellonella* larvae infected with *P. aeruginosa*, isolate these surviving cells, and characterise their phenotype and genotype. **Methods**: Infected larvae were treated with effective doses of either ceftazidime or meropenem. Despite this, surviving *P. aeruginosa* colonies were isolated from living larvae, and antibiotic killing, fitness, virulence, antibiotic resistance and the whole genome sequence of a selection of these isolates were compared with their original parent strains. **Results**: The surviving isolates had an increased minimum duration to kill 99% of the population (MDK_99_) upon exposure to ceftazidime or meropenem and decreased growth rates in culture, but they showed no change to the MIC or virulence—consistent with an antibiotic-tolerant phenotype. Long-read genome sequencing of selected isolates revealed only one single nucleotide polymorphism (SNP) within *bkdB*, encoding the lipoamide acyltransferase component of the branched-chain α-keto acid dehydrogenase, present in two independent isolates. However, time-kill assays with ceftazidime of *bkdB* knockout strains showed no significant change in the MDK_99_. Concomitant with the antibiotic-tolerant phenotype, many of the isolates also had a reduced rate of killing when exposed to heat stress. **Conclusions**: *P. aeruginosa* cells that survived antibiotic therapy in vivo were found to be antibiotic-tolerant and thermotolerant but not antibiotic-resistant and had reduced growth rates under optimal conditions but unchanged virulence. In the absence of a convincing genetic explanation, the co-induction of enhanced thermotolerance with antibiotic tolerance indicated that both are conferred by a heritable phenotypic mechanism.

## 1. Introduction

*P. aeruginosa* is a Gram-negative pathogen commonly causing a range of nosocomial infections including surgical site infections, urinary tract infections and bacteraemias, but, most commonly, pneumonia [1]. Typically, *P. aeruginosa* affects immunocompromised patients and is the cause of up to 20% of cases of ventilator-associated pneumonia [2], with an associated mortality rate greater than 40% [3]. Furthermore, infection with *P. aeruginosa* is associated with poorer outcomes and higher mortality rates for urinary tract infections, surgical site infections and bacteraemias compared with other pathogens [2]. For patients with cystic fibrosis (CF), *P. aeruginosa* is the most common pathogen associated with the condition and is the principal cause of mortality [4]. Chronic infection with the pathogen in these patients results in declining lung function, such that early, aggressive antibiotic therapy is recommended to prevent lung disease [5].

The prevalence of *P. aeruginosa* infections means that antibiotic-resistance of this pathogen is becoming a serious problem in clinical settings. For example, a study from the USA revealed that 18% of isolates from intensive care units were shown to be multi-drug resistant (MDR)—resistant to three or more antibiotic classes—with 26%, 26% and 27% resistance to carbapenems, extended-spectrum cephalosporins and fluoroquinolones, respectively [2]. Less commonly discussed than antibiotic resistance is the potential role of antibiotic tolerance in treatment failure in the clinical setting [6]. Antibiotic tolerance describes the ability of an entire bacterial population to survive transient exposure to an otherwise lethal concentration of antibiotic without exhibiting resistance (i.e., an elevated minimum inhibitory concentration [MIC]) [7]. This tolerance can be either phenotypic (occurring in response to environmental conditions, for example) or genotypic. The bacterial phenomenon of persistence can be considered a subtype of tolerance, whereby only a small sub-group of bacteria within a population exhibit tolerance (reviewed in [8,9]). Many studies have described the emergence of antibiotic-tolerant and persister cells in vitro [10,11], including in *P. aeruginosa* [12]. Notably, evidence is accumulating that such cells also arise in vivo [13,14,15,16,17], and their potential role in causing recalcitrant, or recurring, infections is being investigated. For example, Schumacher et al. [18] showed that *Escherichia coli* persister cells were selected by antibiotic therapy in recurrent urinary tract infections in patients. Also, antibiotic treatment of CF patients with chronic airway infection results in increased isolation of persister cells of *P. aeruginosa* that have been hypothesised to be responsible for maintaining the infection [13]. Worryingly, evidence has been presented that antibiotic tolerance can promote the evolution of antibiotic resistance [16,19], possibly compounding the problem of antibiotic treatment failure.

The mechanisms that underpin antibiotic tolerance are not fully understood but many have been associated with a slow-growing phenotype that can be induced by exposure to stress [6,9]. For example, the stringent stress response and increased levels of the key mediator guanosine pentaphosphate ([(p)ppGpp] [20]; the SOS response [21]; and drop in intracellular ATP levels) [22]. These stress responses are triggered by a range of stress factors that have been shown to induce tolerance or persister formation, including nutrient starvation, oxidative, acidic and osmotic stress and antibiotics. Bacteria are exposed to many of these stress factors in the host [9,23,24].

Previous studies in this lab that utilized the *Galleria mellonella* invertebrate infection model identified novel treatments that conferred significant therapeutic benefit against infections with MDR strains of *P. aeruginosa* [25,26,27,28]. A common observation in all these studies was that, despite the various treatments being highly efficacious and providing excellent therapeutic benefits, none were able to eliminate all infecting *P. aeruginosa*. This led to the hypothesis that the otherwise successful therapies mentioned above could be inducing the formation of a small sub-population of surviving tolerant or persister cells that did not kill the larvae.

The aims of this study were to induce the formation of antibiotic-tolerant and/or persister cells in vivo and to isolate these cells and describe their phenotype and genotype compared with the original parent strains to gain insight into mechanisms of antibiotic tolerance/persistence.

## 2. Results

### 2.1. Susceptibility and Killing of P. aeruginosa Strains by Ceftazidime and Meropenem

Two strains of *P. aeruginosa* were used in this study: NCTC13437, a clinical MDR isolate that is resistant to β-lactams, quinolones and aminoglycosides; and, for comparison, PAO1, an antibiotic-susceptible reference strain. The MIC of meropenem against *P. aeruginosa* PAO1 and NCTC13437 was 0.5 mg/L and 128 mg/L, respectively, while the MIC of ceftazidime was 1 mg/L and >512 mg/L, respectively. The European Committee on Antimicrobial Susceptibility Testing (EUCAST) defines resistance to ceftazidime and meropenem as MICs of >8 mg/L for both [29]. Therefore, by these definitions, NCTC13437 is resistant to both drugs, whilst PAO1 is sensitive.

**Figure 1:**
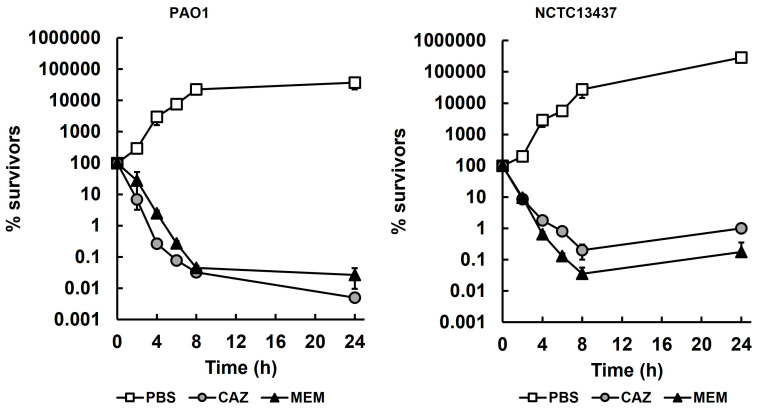
Time-kill assays of *P. aeruginosa* PAO1 and NCTC13437 exposed to meropenem and ceftazidime at 37 °C. Antibiotic concentrations were 4× MIC, apart from MEM with NCTC13437 at 1× MIC (PAO1—CAZ—4 mg/L, MEM—2 mg/L; NCTC 13437—CAZ—2500 mg/L, MEM—128 mg/L). The starting inoculum was approximately 1.0 × 10^7^ cfu/mL. % survival following antibiotic exposure was measured at 0, 2, 4, 6, 8 and 24 h and compared with exposure to sterile PBS. The experiments were completed in triplicate and plotted as the mean with error bars representing ± standard error (SEM). MEM, meropenem; CAZ, ceftazidime.

Time-kill assays to determine the killing kinetics of meropenem and ceftazidime against both *P. aeruginosa* strains are shown in Figure 1. The antibiotics were used at 4× their respective MIC, apart from meropenem, which was used at its MIC for NCTC13437 (2× and 4× MIC were observed to kill the cells too rapidly for accurate quantification). As expected, the untreated control populations of both strains, exposed to PBS, were not inhibited and reached the stationary phase after 24 h. In contrast, exposure to either meropenem or ceftazidime resulted in a rapid loss in the viability of both strains up to 8 h, but, with both antibiotics and both strains, exposure beyond 8 h resulted in little further loss in viability (a small increase in viable numbers in the presence of both antibiotics was observed with NCTC13437). In all cases, after 24 h exposure, a small population of surviving bacteria were detected at approximately 2–3 log_10_ cfu/mL.

The minimum durations to kill 99% (MDK_99_) of *P. aeruginosa* PAO1 and NCTC13437 treated with meropenem or ceftazidime, derived from the killing kinetics shown in Figure 1, are presented in Table 1. The MDK_99_ values shown were used in subsequent studies for comparison with isolates from in vivo studies.

### 2.2. G. mellonella Larvae Infected with P. aeruginosa and Treated with Meropenem or Ceftazidime Show Enhanced Survival Despite the Persistence of Low Numbers of Infecting Bacteria

**Figure 2:**
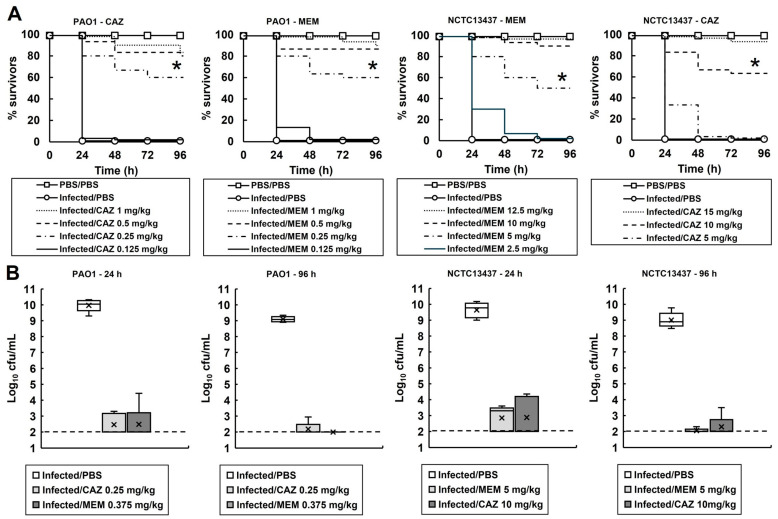
(**A**) Dose-dependent efficacy of CAZ or MEM against *G. mellonella* larva infected with *P. aeruginosa* PAO1 or NCTC13437 at 37 °C. Larvae were infected with 2.5 × 10^3^ cfu/mL of either strain. A single dose of the antibiotic treatments was administered 2 h p.i. An uninfected control group was included, with larvae sham-infected with sterile PBS and treated with sterile PBS at 2 h p.i. The infected control group was infected with either strain of *P. aeruginosa* followed by treatment with sterile PBS 2 h p.i. * indicates significantly enhanced survival compared to the infected control treated with PBS (*p* < 0.05, log rank test); *n* = 30 (pooled from duplicate experiments). (**B**) Box plots of the internal bacterial burden of *G. mellonella* larvae infected with *P. aeruginosa* NCTC13437 or PAO1 and treated with a single dose of PBS, CAZ or MEM measured after 24 and 96 h p.i. at 37 °C. Doses of antibiotics were selected that showed significant therapeutic benefit in A. A group of 25 larvae were infected with 2.5 × 10^3^ cfu/mL of either strain treated with a single dose of PBS, CAZ or MEM at 2 h p.i., as shown. At 24 and 96 h, five caterpillars from each experimental condition were chosen at random, and the internal burden was assessed from each. ‘X’ indicates the mean, the bar indicates the median and the error bars show the highest and lowest values within the dataset. Outlier data is shown as independent points. For each strain, the antibiotic treatment showed a significant reduction in bacterial burden compared with PBS mock treatment (*p* < 0.05, the Mann–Whitney U-test; *n* = 5). PBS—phosphate buffered saline; MEM—meropenem; CAZ—ceftazidime.

To determine if the small surviving population of bacteria observed in vitro (Figure 1) was apparent during treatment of *P. aeruginosa* infections in vivo, the effect of treatment with meropenem or ceftazidime on the bacterial burden and survival of infected *G. mellonella* was measured (Figure 2).

The effect of increasing doses of either meropenem or ceftazidime on the survival of larvae infected with either *P. aeruginosa* PAO1 or NCTC13437 is shown in Figure 2A. Administration of either antibiotic resulted in dose-dependent survival of the larvae infected with either *P. aeruginosa* strain, compared with larvae sham-treated with PBS. The dose of each antibiotic required to confer enhanced survival of *G. mellonella* larvae infected with either *P. aeruginosa* PA01 or NCTC13437 reflected the MIC values. For example, the higher MIC values of *P. aeruginosa* NCTC13437 for ceftazidime and meropenem correlated with the much higher doses of antibiotic required to confer similar rates of survival in vivo when compared with larvae infected with *P. aeruginosa* PAO1.

From these data, doses of each antibiotic that conferred significant survival of larvae were selected for each *P. aeruginosa* strain to measure the effect on the internal burden of infecting bacteria: PAO1—0.25 and 0.375 mg/kg and NCTC13437—10 and 5 mg/kg of ceftazidime and meropenem, respectively (Figure 2B). The internal larval burden of infecting *P. aeruginosa* was measured at 24 and 96 h p.i., and the effect of treatment with ceftazidime or meropenem was compared with larvae sham-treated with PBS. After just 24 h, PBS-treated larvae all died, and high numbers of infecting *P. aeruginosa* were recovered. In contrast, with antibiotic-treated larvae, low numbers of infecting *P. aeruginosa* were recovered at both 24 and 96 h p.i. from living larvae (except for larvae infected with *P. aeruginosa* PAO1 and treated with meropenem, where no surviving bacteria were recovered at 96 h p.i.). There was a clear correlation between the survival of infected larvae and the numbers of infecting bacteria that were recovered, but in most cases, bacteria were not eliminated and living larvae still contained viable *P. aeruginosa*. Therefore, like the data obtained in vitro (Figure 1), the in vivo infection and antibiotic treatment of larvae also resulted in the presence of a small population of surviving cells compared with those sham-treated with PBS.

### 2.3. Cells Derived from P. aeruginosa PAO1 and NCTC13437 and Isolated as Single Colonies from Antibiotic-Treated Larvae Display Phenotypic Differences Compared to Their Parent Strains

To study the cells remaining after in vivo treatment, a single colony was isolated from each of five living larvae that had been infected with *P. aeruginosa* PAO1 or NCTC13437 and treated with either a single dose of ceftazidime or meropenem. All colonies were isolated after 96 h p.i., except for larvae infected with *P. aeruginosa* PAO1 and treated with meropenem, which were isolated from larvae at 24 h p.i. (in this case, no surviving bacteria were recovered after 96 h p.i.). The colonies were picked, inoculated into MHB broth, and cultured overnight prior to the addition of glycerol and storage at −70 °C. These stored cultures were then used to inoculate fresh MHB and cultured again overnight to produce cells for subsequent phenotypic/genotypic analysis. Notably, this meant that the subsequent experimentation was carried out on cells that had been cultured twice under optimal conditions after their initial isolation from the infected, antibiotic-treated larvae.

A preliminary phenotypic screen of the 25 isolates recovered from larvae infected with both strains and treated with both antibiotics was performed. The comparisons of their growth rate, MDK_99_, recovery at 24 h, MIC, and virulence with the untreated parent strains of *P. aeruginosa* NCTC13437 and PAO1 are shown in Supplementary Tables S1 and S2, respectively. Phenotypic differences can be observed between the isolates derived from each *P. aeruginosa* strain that was exposed to the two different antibiotics in the larvae when compared with the untreated parent strains. However, each group of five independent isolates derived from the same strain and isolated after exposure to the same antibiotic treatment displayed broadly consistent phenotypes. For example, the isolates derived from *P. aeruginosa* NCTC13437 exposed to meropenem generally displayed large increases in their MDK_99_, more so than any other group (>55%), but only small decreases in the growth rate were observed, and only one out of the five isolates was able to fully recover after 24 h. The differences in MDK_99_ and growth rate of the recovered isolates after ceftazidime exposure were consistently small, but, in contrast, all were able to fully recover and grow after 24 h (Supplementary Table S1). Isolates derived from *P. aeruginosa* PAO1 exposed to ceftazidime all displayed a consistent decrease in growth rate of around 45–55% and a consistent increase in MDK_99_ of approximately 50%. Exposure to meropenem, however, resulted in consistently smaller decreases in growth rate and smaller increases in MDK_99_. None of the strains isolated after exposure to meropenem were able to recover and grow after 24 h, and only one of those exposed to ceftazidime was able to fully recover (Supplementary Table S2). Notably, none of the recovered isolates derived from either strain, or after exposure to either antibiotic, showed any change in their respective MIC values for each antibiotic. The degree of virulence, measured after reinfection of *G. mellonella* larvae, also remained unchanged (with the single exception of *P. aeruginosa* NCTC13437 derived isolate 7, where reinfection resulted in a 27% increase in the survival of larvae compared to infection with the untreated parent strain).

**Figure 3:**
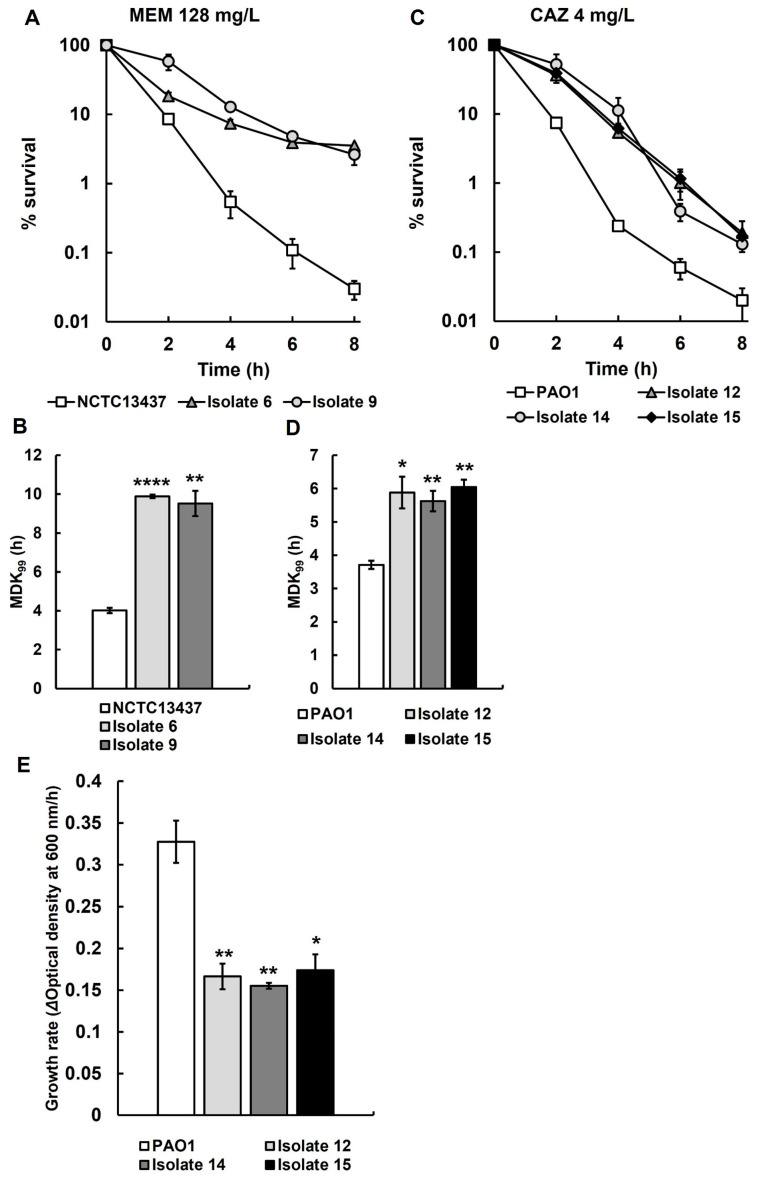
Time-kill assay with MEM (128 mg/L) for recovered isolates 6 and 9 from larvae infected with *P. aeruginosa* NCTC13437 after treatment with MEM (**A**), and time-kill assay with CAZ (4 mg/L) of recovered isolates 12, 14 and 15 from larvae infected with *P. aeruginosa* PAO1 after treatment with CAZ (**C**). The starting inoculum of each strain was approximately 1.0 × 10^7^ cfu/mL. % survival of each strain following antibiotic exposure was measured at 0, 2, 4, 6 and 8 h and compared with the untreated parent strains. The experiments were completed in triplicate and plotted as the mean with error bars representing ± standard error (SEM). Minimum duration to kill (MDK_99_) calculated from the data presented in A and C, of recovered isolates 6 and 9 exposed to MEM (128 mg/L) (**B**) and recovered isolates 12, 14 and 15 exposed to CAZ (4 mg/L) (**D**), compared with their untreated parent strains. (**E**) The growth rate of recovered isolates 12, 14 and 15 was compared with their untreated parent strain *P. aeruginosa* PAO1. Significant difference from the untreated parental strain(s) was determined using unpaired two-tailed parametric *t*-tests: * *p* < 0.05, ** *p* < 0.01, and **** *p* < 0.0001). MEM—meropenem; CAZ—ceftazidime.

The isolates that showed an increase in MDK_99_ and a reduced growth rate without a change to the MIC are consistent with an antibiotic-tolerant phenotype. Of the 25 recovered isolates initially screened in Supplementary Tables S1 and S2, those displaying the most antibiotic-tolerant phenotypes were selected, and antibiotic time-kill assays and growth rate determination were carried out in triplicate (Figure 3). Time-kill assays of isolates 6 and 9, derived from *P. aeruginosa* NCTC13437 exposed to meropenem in vivo, show significantly reduced rates of killing by meropenem (Figure 3A) and significantly increased MDK_99_ times (Figure 3B) when compared with the parent strain. Similarly, isolates 12, 14 and 15, derived from *P. aeruginosa* PAO1 exposed to ceftazidime in vivo, show significantly reduced rates of killing by ceftazidime (Figure 3C) and significantly increased MDK_99_ times (Figure 3D) when compared with the parent. Isolates 12, 14 and 15 all showed significant reductions in growth rate compared to untreated *P. aeruginosa* PAO1 in MHB at 37 °C (Figure 3E) but, in contrast, isolates 6 and 9 did not show any significant change in growth rate in MHB compared with untreated *P. aeruginosa* NCTC13347.

### 2.4. Short-Read Whole Genome Sequencing of P. aeruginosa Isolates Recovered After Antibiotic Exposure In Vivo Detects No Changes When Compared with the Untreated Parental Strains

The antibiotic-tolerant phenotypes that were identified in the recovered isolates of *P. aeruginosa* in vivo were present after two subsequent rounds of growth in vitro after their initial isolation from infected and antibiotic-treated larvae. This implies that the antibiotic-tolerant phenotypes these isolates possess are heritable. To determine if any genetic changes could be identified that could account for the antibiotic-tolerant phenotypes, the same five tolerant isolates characterised above were selected for whole genome sequencing, in addition to their original parent strains, for comparison.

From short-read whole genome sequencing data of the recovered isolates 6, 9, 12, 14 and 15, variant calling was undertaken versus their respective untreated parent strains. Variant calling aligned and compared the assembled reads from the recovered isolates with their respective untreated parent strains that represented the reference genomes to identify genetic differences. No variants were found in the recovered isolates relative to the assembly of their parent strains. To improve the likelihood of identifying potential mutations that could explain the antibiotic phenotypes shown by the recovered isolates, further experimentation was undertaken.

### 2.5. G. mellonella Larvae Infected with P. aeruginosa PAO1 and Treated with Three Doses of Ceftazidime Show Enhanced Survival Despite the Persistence of Small Numbers of Infecting Bacteria

As no mutations in the recovered isolates were identified by short-read whole genome sequencing, an attempt to isolate surviving isolates was repeated following multiple exposure to an antibiotic in vivo, with the rationale that increased exposure to the antibiotic stress could enhance the likelihood of isolating survivors with mutations, which could inform the basis of the antibiotic-tolerant phenotype. Also, it was decided that any recovered isolates would be subjected to long-read genome sequencing to improve the probability of identifying any mutations accurately.

**Figure 4:**
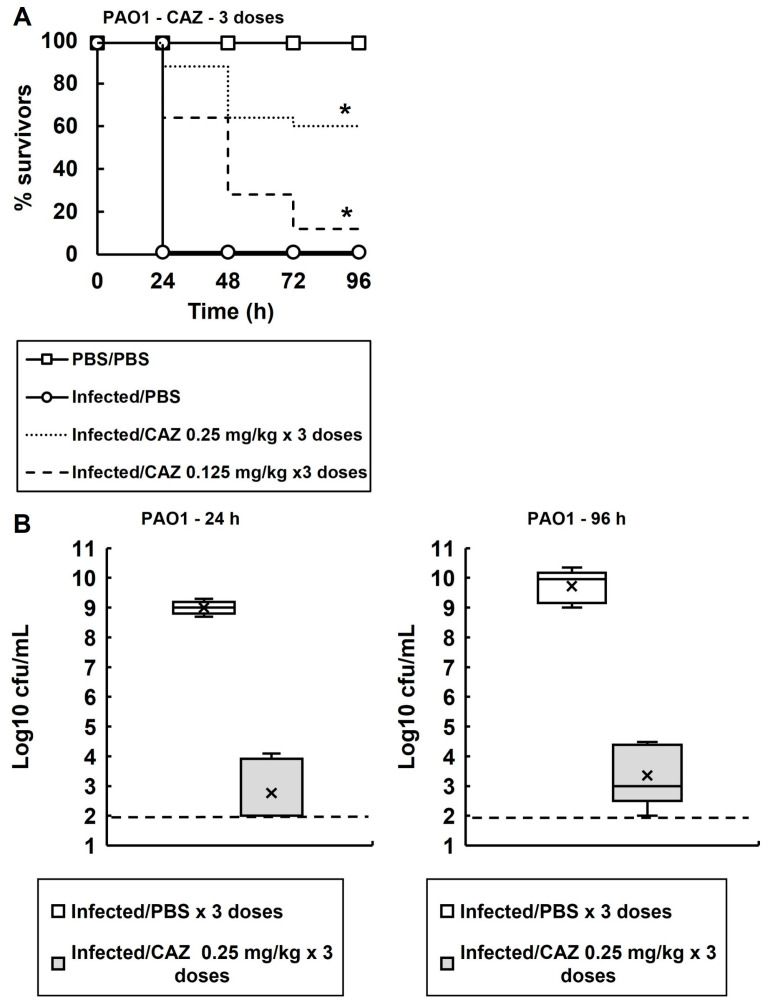
(**A**) Dose-dependent efficacy of CAZ against *G. mellonella* larva infected with *P. aeruginosa* PAO1 at 37 °C. Larvae were infected with 2.5 × 10^3^ cfu/mL of *P. aeruginosa* cells. Three doses of CAZ (0.125 or 0.25 mg/kg) were administered at 2, 24 and 48 h p.i. An uninfected control group was included with larvae sham-infected with sterile PBS and treated with three doses of sterile PBS at 2, 24 and 48 h p.i. The infected control group was infected with *P. aeruginosa* PAO1 followed by treatment with three doses of sterile PBS. * Indicates significantly enhanced survival compared to the infected control treated with PBS (*p* < 0.05, log rank test); *n* = 30 (pooled from duplicate experiments). (**B**) Box plots of the internal bacterial burden of *G. mellonella* larvae infected with *P. aeruginosa* PAO1 and treated with three doses of PBS or CAZ (0.25 mg/kg) measured 24 and 96 h p.i. at 37 °C. The dose of CAZ selected showed significant therapeutic benefit in A. A group of 25 larvae were infected with 2.5 × 10^3^ cfu/mL of *P. aeruginosa* PAO1 and, 2, 24 and 48 h p.i., were treated with PBS or CAZ. At 24 and 96 h, five healthy caterpillars from each experimental condition were chosen at random and the internal burden was assessed from each. ‘X’ indicates the mean, the bar indicates the median and the error bars show the highest and lowest values within the dataset. Outlier data is shown as independent points. For each strain, the antibiotic treatment showed a significant reduction in bacterial burden compared with PBS mock treatment (*p* < 0.05, the Mann–Whitney U-test; *n* = 5). PBS—phosphate buffered saline; CAZ—ceftazidime.

*G. mellonella* larvae were infected with *P. aeruginosa* PAO1 and exposed to multiple doses of ceftazidime (Figure 4A). Dose-dependent efficacy of ceftazidime was observed, and three doses of 0.25 mg/kg at 2, 24 and 48 h p.i. resulted in approximately 60% survival of infected larvae after 96 h. Similar to previous observations (Figure 2), the above dosing regimen resulted in a drastic reduction in the numbers of viable bacteria recovered from the infected larvae, at 24 and 96 h p.i., compared with larvae sham-treated with PBS (Figure 4B). As before, despite the dosing of ceftazidime significantly enhancing the survival of the larvae, small numbers of bacterial survivors were still recovered after 24 and 96 h.

### 2.6. P. aeruginosa PAO1 Isolates Recovered from Infected Larvae Treated with Three Doses of Ceftazidime Have Antibiotic-Tolerant Phenotypes Compared to Their Untreated Parent Strain

As before, a single surviving colony was isolated from each of five living larvae that had been infected with *P. aeruginosa* PAO1 and treated with three doses of ceftazidime. As previously, all the colonies were isolated after 96 h p.i., inoculated into MHB broth, and cultured overnight prior to the addition of glycerol and storage at −70 °C. These stored cultures were then used to inoculate fresh MHB and cultured again overnight to produce cells for subsequent phenotypic/genotypic analysis.

Comparison of the growth rate, minimum duration to kill 99% of the population (MDK_99_), recovery at 24 h, MIC and virulence of all isolates recovered from infected larvae with the untreated parent strains of *P. aeruginosa* PAO1 is shown in Table 2. The group of five independent recovered isolates displayed broadly consistent antibiotic-tolerant phenotypes. For example, they all displayed a consistent decrease in growth rate of around 20–25% and a consistent increase in MDK_99_ for ceftazidime of approximately 40%. None of the isolates were able to recover and grow after 24 h. As with the previously recovered isolates, none showed any change in their MIC values for ceftazidime, and the degree of virulence measured after reinfection of *G. mellonella* larvae also remained unchanged.

**Figure 5:**
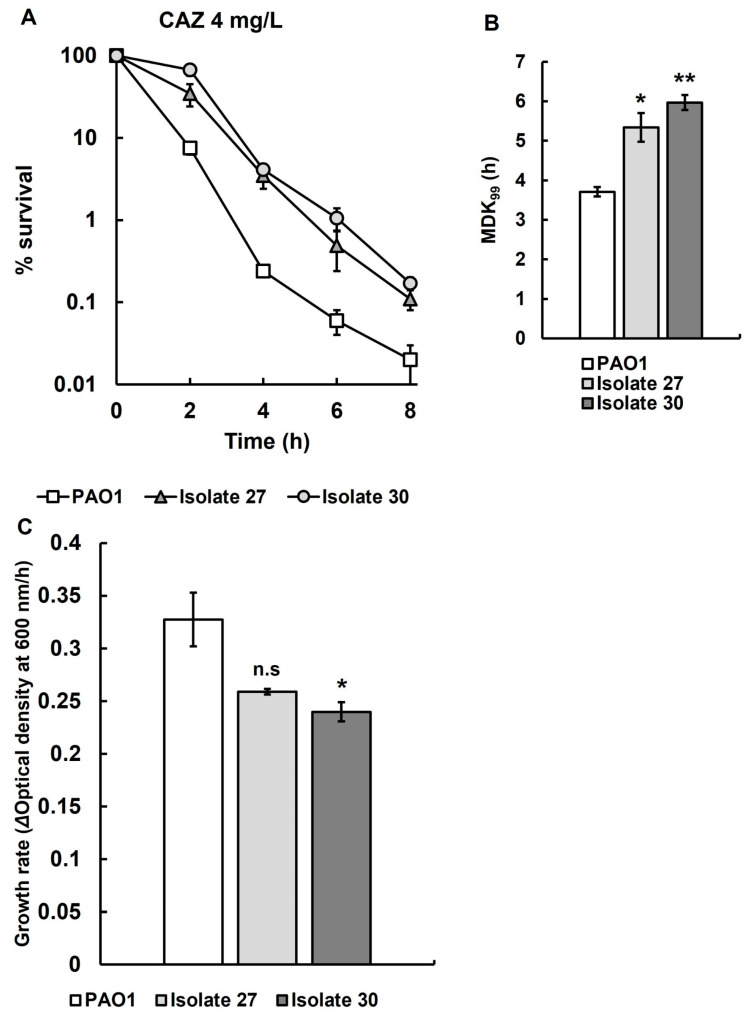
(**A**) Time-kill assay with CAZ (4 mg/L) of recovered isolates 27 and 30 from larvae infected with *P. aeruginosa* PAO1 after treatment with three doses of CAZ (0.25 mg/kg) 2, 24 and 48 h p.i. The starting inoculum of *P. aeruginosa* PAO1 was approximately 1.0 × 10^7^ cfu/mL. % survival following antibiotic exposure was measured at 0, 2, 4, 6 and 8 h and compared with the untreated parent strain. The experiments were completed in triplicate and plotted as the mean with error bars representing ± standard error (SEM). (**B**) Minimum duration to kill (MDK_99_), calculated from the data of recovered isolates 27 and 30 exposed to CAZ (4 mg/L) presented in A, compared with the untreated parent strain. (**C**) The growth rates of recovered isolates 27 and 30 were compared with their untreated parent strain *P. aeruginosa* PAO1. Significant difference to the untreated parental strain(s) was determined using unpaired two-tailed parametric *t*-tests: * *p* < 0.05, ** *p* < 0.01). n.s—no significant difference; CAZ—ceftazidime.

Recovered isolates 27 and 30 were selected for more detailed study, and antibiotic time-kill assays and growth rate determination were carried out in triplicate (Figure 5). Time-kill assays of recovered isolates 27 and 30 show significantly reduced rates of killing by ceftazidime (Figure 5A) and significantly increased MDK_99_ times (Figure 5B) when compared with the untreated parent strain *P. aeruginosa* PAO1. Recovered isolate 30 showed a significant reduction in growth rate compared to the untreated parent strain in MHB at 37 °C (Figure 5C), but, in contrast, the reduction in growth rate of recovered isolate 27 was not significant when compared with untreated *P. aeruginosa* PAO1.

### 2.7. Long-Read Whole Genome Sequencing of P. aeruginosa PAO1 Isolates Recovered After Exposure to Three Doses of Ceftazidime In Vivo Reveals a SNP in the bkdB Gene Causing a Missense Variation

The antibiotic-tolerant isolates 27 and 30 were subjected to long-read whole genome sequencing, with their original parent strain for comparison, to determine if any genetic changes had occurred. Genome analysis showed no significant genomic rearrangements or other structural variations compared to the parent, *P. aeruginosa* PAO1, other than a 6-bp indel in a low-complexity region of *pscP*, which alters the length of a 22-amino acid proline-threonine repeat. Variant calling of isolates 27 and 30 revealed a single nucleotide polymorphism (SNP) representing a single nucleotide substitution of cytosine for thymine present in both independent isolates within a gene encoding the lipoamide acyltransferase component of the branched-chain α-keto acid dehydrogenase (*bkdB*) (Table 3). In isolate 27, 70% of the population harboured the mutation, while 30% matched the reference genome of PAO1. In Isolate 30, 51% of the population harboured the mutation and 49% matched the reference genome of *P. aeruginosa* PAO1.

### 2.8. Mutant P. aeruginosa Strains with a bkdB Gene Knockout Do Not Display an Antibiotic-Tolerant Phenotype Compared to the Parent P. aeruginosa MPAO1

To determine if the mutations identified in the recovered isolates 27 and 30 confer antibiotic tolerance due to a loss of function of BkdB, *P. aeruginosa* strains PW4821 and PW4822, that each harboured an independent *bkdB* gene knockout by transposon insertion, were purchased from the Salipante lab (https://sites.google.com/uw.edu/salipante-lab, accessed on 11 May 2025) in addition to their parent strain *P. aeruginosa* MPAO1. Colony PCR was performed using flanking forward and reverse primers to confirm that no *bkdB* was present in both the PW4821 and PW4822 strains, indicating gene knockout. In addition, colony PCR was performed using the forward flanking primer paired with a transposon specific primer (lacZ-138) to confirm the presence of the intended transposon in *bkdB* (Supplementary Table S3). These primers confirmed the presence of the intended transposon insertion within *bkdB* in both knockout strains but not the parent strain.

Time-kill assays with ceftazidime, and the growth rate of both *bkdB* knockout strains (PW4821 and PW4822), were measured and compared with their parent strain *P. aeruginosa* MPAO1 (Supplementary Table S4). Neither *bkdB* knockout strain showed a significant change in killing kinetics by ceftazidime compared to the parent strain, and the MDK_99_ values were not significantly different. Furthermore, there was no significant difference in growth rate between both *bdkB* knockout strains and the parent strain. Therefore, loss of function of *bkdB* does not account for the antibiotic-tolerant phenotype observed in isolates 27 and 30 recovered in vivo after exposure to ceftazidime.

### 2.9. The Recovered Antibiotic-Tolerant P. aeruginosa Isolates Are Significantly More Thermotolerant than Their Untreated Parent Strains

**Figure 6:**
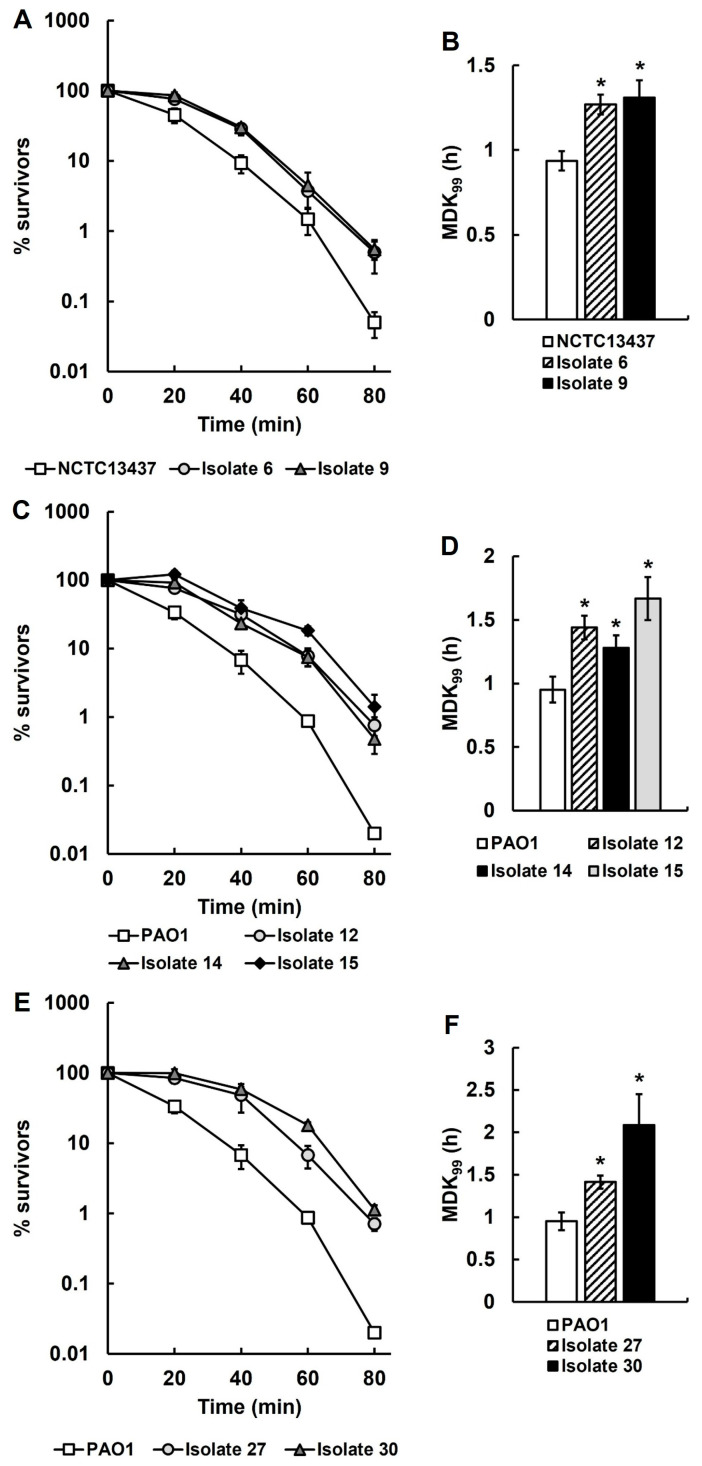
Time-kill (**A**,**C**,**E**) and minimum duration to kill (MDK_99_) (**B**,**D**,**F**) assays at 50 °C of *P. aeruginosa* recovered isolates from infected larvae treated with a single dose of MEM (isolates 6 and 9; (**A**,**B**)) or CAZ (isolates 12, 14 and 15; (**C**,**D**)), or three doses of CAZ (isolates 27 and 30; (**E**,**F**)), compared with untreated parental *P. aeruginosa* PAO1 or NCTC13437. The starting inoculum of each *P. aeruginosa* strain was approximately 1.0 × 10^7^ cfu/mL. % survival of the recovered isolates was measured after 0, 20, 40, 60 and 80 min exposure at 50 °C and compared with the respective untreated parent strains. The experiments were completed in triplicate and plotted as the mean with error bars representing ± standard error (SEM). The MDK_99_ at 50 °C was calculated from the data presented in the time-kill curves and compared with their respective untreated parent strains. Significant differences in the MDK_99_ to the untreated parental strain(s) was determined using non-linear parametric *t*-tests: * *p* < 0.05.

Genome sequencing of several recovered isolates only identified one SNP that could account for their antibiotic-tolerant phenotype. However, knockout of the gene harbouring this SNP had no effect on antibiotic tolerance. As an alternative explanation, it was hypothesised that the antibiotic-tolerant phenotype could be conferred by a general stress response in the recovered isolates induced by exposure to both antibiotic therapy and the host immune response. This general stress response could confer tolerance to other stress conditions such as heat. Heat tolerance assays were conducted to determine the killing kinetics of each of the recovered isolates at 50 °C and compared with their respective untreated parent strains (Figure 6). Thermotolerance assays of isolates 6 and 9, derived from *P. aeruginosa* NCTC13437 exposed to meropenem in vivo, show significantly reduced rates of killing at 50 °C (Figure 6A) and significantly increased MDK_99_ times (Figure 6B) when compared with the untreated parent strain. Similarly, recovered isolates 12, 14 and 15, derived from *P. aeruginosa* PAO1 exposed to ceftazidime in vivo, show significantly reduced rates of killing at 50 °C (Figure 6C) and significantly increased MDK_99_ times (Figure 6D) when compared with the untreated parent. This result was also replicated with the isolates 27 and 30 derived from *P. aeruginosa* PAO1 exposed to three doses of ceftazidime in vivo (Figure 6E,F). To conclude, antibiotic-tolerant isolates of *P. aeruginosa* selected in vivo consistently display enhanced thermotolerance.

## 3. Discussion

In this work, *P. aeruginosa* cells were isolated from *G. mellonella* larvae that survived an otherwise lethal infection after successful treatment with either ceftazidime or meropenem. Colonies were recovered from larvae infected with either *P. aeruginosa* PAO1 or an MDR strain, *P. aeruginosa* NCTC13437 and treated with a single dose of either ceftazidime or meropenem. Colonies were also independently isolated from larvae infected with *P. aeruginosa* PAO1 after treatment with three doses of ceftazidime. The strains were isolated as single colonies recovered from the antibiotic-treated larvae and were subsequently identified to be antibiotic-tolerant compared with their respective untreated parent strains. *G. mellonella* has been employed previously to study antibiotic persister cells of *Acinetobacter baumannii* [30], and another successful example of the use of alternative in vivo models is *Drosophila melanogaster* to study antibiotic-tolerant cells of *Escherichia coli* [31].

Notably, in this work, antibiotic-tolerant cells were also isolated from infections with the antibiotic-resistant strain *P. aeruginosa* NCTC13437, demonstrating that antibiotic-resistance and tolerance are mutually exclusive and can both contribute to pathogen survival in the presence of antibiotics. Antibiotic tolerance was inferred due to increased MDK_99_ values in the presence of antibiotics, reduced growth rates under optimal conditions and no change in the MIC values for the antibiotics used. The unchanging MIC value indicates that the cells had not acquired resistance to the antibiotics (or additional resistance, in the case of NCTC13437), and the reduced growth rate, alongside the inability to recover and grow in the presence of the antibiotic, correlates with a host of studies that have linked surviving cells to reduced fitness and dormancy (reviewed in [9]). If the survivors reduce their overall metabolic activity—whereby essential metabolic pathways such as peptidoglycan synthesis, that is targeted by ceftazidime and meropenem used in this work, are downregulated—this could explain reduced rates of killing by the antibiotics that rely on targeting these pathways for their inhibitory action [32]. This conclusion is supported by a study that uncoupled growth rate from metabolic activity and showed that the inhibitory activity of antibiotics is dependent on the metabolic state of bacteria rather than growth rate [33]. Thermotolerance assays also revealed that many of the antibiotic-tolerant survivors also had a reduced rate of killing when exposed to heat stress. This implies that the mechanism conferring antibiotic tolerance is non-specific and induces a stress response that confers both heat and antibiotic tolerance (reviewed in [34]). Perhaps contradicting this, the antibiotic-tolerant phenotypes measured in the survivors were retained after the isolates were subsequently cultured twice in vitro in the absence of any antibiotics, implying that the mechanism(s) conferring the antibiotic tolerance are heritable. However, short-read whole genome sequencing was unable to identify a genetic alteration that could account for the antibiotic-tolerant phenotype observed. Due to the reduced coverage of short-read whole genome sequencing, and its inability to detect structural variants compared with long-read sequencing, it is possible that significant mutations were not detected. Also, the small number of isolates that were sequenced could mean that other significant mutations were not detected. In contrast, long-read whole genome sequencing of survivors isolated from infected larvae exposed to a triple dose of ceftazidime did identify a single SNP in the gene encoding the lipoamide acyltransferase component (*bkdB*) of the branched-chain alpha-keto acid dehydrogenase complex (BKD). The BKD complex is a multi-enzyme complex responsible for catalysing the conversion of branched-chain alpha-keto acids to branched-chain acyl-CoA and CO_2_ [35,36]. This reaction is involved in the catabolism of branched-chain amino acids such as leucine, isoleucine and valine to build branched-chain fatty acids (BCFA) that are required to maintain the bacterial membrane and provide a source of energy. A single nucleotide substitution of cytosine for thymine in *bkdB* was identified in two independent antibiotic-tolerant in vivo isolates and thus could account for the tolerant phenotype. The substitution would result in a missense variation in the protein, substituting aspartic acid for asparagine at position 232. The presence of the mutation in each of the independent isolates was mixed, with the sequence of isolates 27 and 30 showing 30 and 48% of the cells had reverted to the parent *P. aeruginosa* PAO1 sequence, respectively. This could indicate a potential fitness cost of the mutation and imply a role in conferring antibiotic tolerance. Nonetheless, phenotypic analysis of two *P. aeruginosa* MPAO1 strains containing independent transposon knockouts of *bkdB* did not reveal any antibiotic-tolerant phenotypes suggesting that, if the mutation in *bkdB* is responsible for the antibiotic-tolerant phenotypes observed in isolates 27 and 30, it does this without inactivation of gene function. However, a change in activity of the enzyme arising from the mutation could still contribute to an antibiotic-tolerant phenotype. To date, the structure of *P. aeruginosa* BkdB has not been experimentally determined but has been described in a structural and functional analysis of the enzyme in the cow, *Bos taurus* [37]. Structural models have been predicted based on the amino acid primary sequence in UniProt (accession number Q9I1M0; https://www.uniprot.org/uniprotkb/Q9I1M0/entry, accessed on 11 May 2025). From this, the Asp-232-Asn mutation detected in this work can be located in the C-terminal domain that is likely responsible for the acyltransferase catalytic activity of the enzyme [36]. Sequence alignment of the primary structure of BkdB from *Bos taurus* and other unrelated species outline the conservation of the active site residues His400 and Asp404 in the C-terminal domain; however, the mutation does not occur in the immediate vicinity of this likely active site and further work is required to determine if the mutation does have any effect on the catalytic activity of the enzyme and, thus, antibiotic tolerance.

Within bacteria, BCFAs are utilised in the bacterial membrane to facilitate structure and fluidity (reviewed in [38]). Speculatively, alteration of the abundance of BCFAs could alter membrane composition, impacting the fluidity and/or structural integrity, which could then influence the ability of antibiotics to gain access to the cell and induce antibiotic tolerance. This mechanism could also explain why the antibiotic-tolerant survivors were also found to have enhanced thermotolerance compared with their parent strains. It is known that bacteria alter membrane composition in response to heat stress in a process known as homeoviscous adaptation [39,40]. The inclusion of BCFAs decreases the melting point of bacterial membranes [41] such that disruption of BCFA synthesis could shift membrane composition towards a heat-tolerant phenotype by increasing the melting point of the membrane.

Alternatively, as alluded to previously, the basis of survival in vivo, and the antibiotic-tolerant phenotype detected in these isolates, may not be due to heritable genetic change but could be phenotypic. The fact that only a single mutation was identified in the antibiotic-tolerant isolates tends to support this conclusion. However, only two isolates were subjected to long-read sequencing, and more definitive conclusions might be drawn if a larger sample size were sequenced and any other important mutations detected. Any processes and/or stress responses induced by exposure to both the antibiotic treatment and larval immune response that confer the antibiotic-tolerant phenotype could remain active, even after being cultured twice more under non-stress conditions in vitro. Future experimentation will investigate if the observed antibiotic-tolerant phenotype disappears with additional rounds of passage, thereby indicating a phenotypic basis for survival, or if the phenotype persists, indicating a heritable genetic basis. Also, it cannot be discounted that the antibiotic-tolerant phenotypic and genotypic changes that were observed in the recovered isolates could be due solely to the infection process and not because of exposure to antibiotic therapy. Future work comparing the phenotype and genotype of isolates from antibiotic-treated larvae with isolates from PBS-treated larvae will provide more definitive insight.

Epigenetic mechanisms are found ubiquitously in bacteria and allow cells to alter their phenotype to produce clonal heterogeneity through a form of gene regulation known as phase variation. Bacterial epigenetics involves modifications of DNA and RNA—principally, methylation of DNA, whereby methyl groups are transferred from S-adenosyl-L-methionine to adenine or cytosine by a group of enzymes called DNA methyltransferases (Mtases). Mtases act on specific DNA sequences and methylate bases such that the methyl group is positioned within the major groove of the DNA helix, where it can bind to, or inhibit, DNA-binding proteins and regulate transcription of various genes that control DNA replication, DNA repair, phase variation switching and virulence [42,43,44]. For example, the Mtases ModA11 and ModA12 can influence susceptibility to cloxacillin and ciprofloxacin in *Neisseria meningitidis*, and the absence of ModA11 sensitises cells to ciprofloxacin and ceftazidime [45]. Notably, epigenetic mechanisms of transcriptional regulation have also been shown to be heritable—where bacteria utilise DNA methylation patterns to pass information on the phenotypic profile of the parent to the daughter cell—and could provide a potential explanation for the persistence of a tolerant phenotype despite the absence of genetic alterations (reviewed in [46,47,48,49]).

To conclude, surviving *P. aeruginosa* cells were isolated from in vivo infections of *G. mellonella* larvae despite successful therapy with the antibiotics meropenem or ceftazidime. Compared with their untreated parent strains, the survivors were found to be antibiotic- and thermotolerant but not resistant and had reduced growth rates under optimal conditions but unchanged virulence. Short-read genome sequencing found no mutations that could account for the antibiotic-tolerant phenotype in surviving cells. Long-read genome sequencing identified only one mutation, an identical single-base change from two independently isolated survivors exposed to successful ceftazidime therapy. The mutation was present in *bkdB*, encoding the lipoamide acyltransferase component of the branched-chain alpha-keto acid dehydrogenase complex. However, deletion of this gene did not induce an antibiotic-tolerant phenotype. Co-induction of enhanced thermotolerance with antibiotic tolerance indicated that both are conferred by a heritable phenotypic mechanism. The results presented show the potential for using *G. mellonella* to study (i) the mechanisms underpinning how antibiotic-tolerant survivors arise in vivo and (ii) new treatment approaches that can prevent the formation of antibiotic-tolerant survivors and reduce the possibility of recurring infections.

## 4. Materials and Methods

### 4.1. Bacteria and Growth Media

Two strains were used: *P. aeruginosa* NCTC13437, a characterised MDR strain (carrying the VEB-1 ESBL and VIM-10 MBL) that is resistant to carbapenems and other β-lactam antibiotics, and quinolones and aminoglycosides by unknown mechanisms [50]; and *P. aeruginosa* PAO1, which was a gift from Dr. Olga Lomovskaya, Rempex Pharmaceuticals, USA. All strains were grown to stationary phase in Mueller–Hinton broth (MHB; Merck, Darmstadt, Germany) at 37 °C with shaking (at 200 rpm) overnight to prepare inocula for antibiotic efficacy testing in vitro and in vivo.

### 4.2. Antibiotics and G. mellonella Larvae

All antibiotics and chemicals were purchased from Sigma–Aldrich Ltd. (Dorset, UK) unless otherwise stated. All sub-stock solutions of meropenem or ceftazidime were prepared in sterile deionized water. *G. mellonella* larvae were obtained from UK Waxworms Ltd. (Sheffield, UK).

### 4.3. Antibiotic Susceptibility Testing

Minimum inhibitory concentrations (MICs) of meropenem and ceftazidime against both *P. aeruginosa* strains were determined in 96-well microplates (Greiner Bio-One, Frickenhausen, Germany) as previously described [51]. Briefly, doubling dilutions of each antibiotic were prepared in MHB and subsequently inoculated with 1.0 × 10^6^ cfu/mL of *P. aeruginosa*. Microplates were incubated at 37 °C, and the MIC was defined as the concentration(s) present in the first optically clear well after 24 h.

### 4.4. In Vitro Time-Kill Assays

The killing kinetics of meropenem and ceftazidime against *P. aeruginosa* PAO1 and NCTC13437 (and all subsequent isolates derived from these parent strains that were isolated from in vivo experiments) were determined in a 96-well microplate. Meropenem and ceftazidime were used at 4× their respective MICs (except where otherwise stated). A *P. aeruginosa* inoculum was used to give a final cell number of 1.0 × 10^6^ cfu/mL. PBS (NaCl 8 g/L, KCl 0.2 g/L, Na_2_HPO_4_ 1.44 g/L, KH_2_PO_4_ 0.245 g/L, pH 7.4) was used in place of the antibiotic for the control. Following inoculation, the microplate was incubated at 37 °C, and samples were removed at 0, 2, 4, 6, 8 and 24 h, serially diluted in MHB, and plated on Nutrient Agar (NA) (Formedium Ltd., Hunstanton, UK). Plates were incubated overnight at 37 °C prior to colony counting and enumeration of bacterial numbers. The detection limit for this assay was 100 cfu/mL. All experiments were performed in triplicate and data plotted as the mean ± SEM. The minimum duration of killing for 99% of the population (MDK_99_) was determined for each individual replicate using the linear regression and interpolation function in GraphPad Prism 6.0.7; only data in the linear range of the MDK were used in the calculation (minimum of three data points), and all *R*^2^ values were >0.9.

### 4.5. G. mellonella Infection Model

*G. mellonella* at their final instar larval stage were kept at room temperature in darkness. Larvae weighing within the range of 250 to 350 mg were selected for each experiment to ensure consistency in subsequent drug administration and were used within 1 week of receipt. Efficacy of meropenem or ceftazidime versus *G. mellonella* larvae infected with the *P. aeruginosa* strains was carried out exactly as described previously [51,52,53]. Briefly, groups of 15 larvae were infected with an inoculum of 2.5 × 10^3^ cfu/mL of *P. aeruginosa* cells. Treatment with a single dose of each antibiotic was administered 2 h post-infection (p.i.). Across all experiments, an uninfected control group treated twice with PBS was included to account for potential injection trauma, and a negative control was included where infected larvae were treated with PBS. For multiple doses of ceftazidime, larvae were treated at 2 h p.i., followed by additional doses at 24 and 48 h p.i. The uninfected control and the negative control groups also received additional doses of PBS at 24 and 48 h. Surviving larvae were counted at 24, 48, 72, and 96 h and classified as alive or dead depending on their visible response to physical stimuli. All experiments were repeated in duplicate using larvae from different batches, and the data from these replicate experiments were pooled to give *n* = 30. Survival data were plotted using the Kaplan–Meier method [54], and comparisons were made between groups using the log-rank test [55].

### 4.6. Burden of P. aeruginosa in G. mellonella Larvae and Isolation of Surviving Cells

To measure the internal burden of *P. aeruginosa* following antibiotic treatment, a standard inoculum size of 2.5 × 10^3^ cfu/mL was used to infect a group of 25 *G. mellonella* larvae. Two hours p.i., larvae were treated with a single dose of meropenem or ceftazidime or triple doses of ceftazidime. At 24 and 96 h p.i., 5 larvae were selected to measure the internal bacterial burden of *P. aeruginosa*. Only healthy larvae with the least melanisation and strongest visual response to physical stimuli, indicating successful antibiotic therapy, were selected. Selected larvae were surface disinfected and anaesthetized in ethanol (100%) prior to being placed in Eppendorf tubes containing 300 µL of PBS. A sterile pestle (Argos Technologies Inc., Eligin, IL, USA) was then used to homogenize the larvae before the addition of a further 300 µL of PBS. The homogenate was then mixed, and 20 µL was removed and serially diluted (ten-fold dilutions) in MHB in a 96-well plate. Dilutions were then plated on Pseudomonas C-N selective agar (agar plates were made following the method provided by Thermo Fisher Scientific containing *Pseudomonas* agar base [Thermo Fisher Scientific, Loughborough, UK]; cetrimide [Glentham Life Sciences, Corsham, UK]; and nalidixic acid [Duchefa Biochemical, Haarlem, The Netherlands]). Agar plates were incubated overnight at 37 °C. This method was carried out on each of the 5 larvae sampled per treatment group. Single colonies of persisters were then picked from plates that represented each of the 5 larvae sampled and used to inoculate 500 µL of MHB in an Eppendorf tube. These were cultured overnight with shaking at 37 °C prior to addition of sterile glycerol (80%) to give a final concentration of 15%, vortexed and stored at −80 °C for subsequent experiments.

### 4.7. Measurement of the MIC, Growth Rate, MDK_99_ and Recovery, Virulence and Thermotolerance of Antibiotic-Treated P. aeruginosa Isolates

The MIC, MDK_99_ and virulence of *P. aeruginosa* isolates that survived antibiotic therapy in vivo were measured as described above and compared with the MIC, MDK_99_ and virulence of their respective parent strains *P. aeruginosa* NCTC13437 or PAO1. Recovery at 24 h was defined as follows: if the log_10_ cfu/mL value in the presence of antibiotic at the 24 h time point in time-kill assays was 2 log_10_ cfu/mL greater than the value measured after the 8 h time point in the same assays, the population was deemed to have recovered.

To measure and compare the growth rate of the isolates that survived antibiotic therapy in vivo with their respective parent strains, overnight cultures were grown in MHB at 37 °C in a shaking incubator. These cultures were used to inoculate 5 mL of fresh MHB to give a starting optical density at 600 nm (OD_600_) of 0.05. These cultures were incubated with shaking at 37 °C and OD_600_ measurements taken every 30 min for 6 h using a Pharmacia Biotech Novaspec II spectrophotometer. Growth rate was calculated as change in OD_600_ over time (hours). Each experiment was performed in triplicate and the mean and ±SEM plotted using Microsoft Excel. Comparison of the thermotolerance at 50 °C of the recovered isolates with their parent strains was measured using a viability assay over 80 min. A sample of 4 mL of MHB was inoculated with a *P. aeruginosa* inoculum to give a starting cell number of 1.0 × 10^6^ cfu/mL and incubated with shaking at 50 °C. A 20 µL sample was taken every 20 min and serially diluted (10-fold dilutions) in fresh MHB in a 96-well plate prior to plating on NA. Plates were then incubated overnight at 37 °C and colonies counted and surviving bacteria enumerated. Each experiment was performed in triplicate and the mean and ±SEM plotted using Microsoft Excel.

### 4.8. Whole Genome Sequencing and Bioinformatic Analysis of P. aeruginosa Antibiotic-Treated Isolates

A selection of *P. aeruginosa* isolates that survived antibiotic therapy in vivo were sequenced to identify genetic differences or mutations that could account for their survival and persister/tolerant phenotype. Isolates for sequencing were selected based on their antibiotic tolerance profiles from time-kill assays and growth rate compared with their respective parent strains. Sequencing was conducted by MicrobesNG (Birmingham Research Park, Birmingham UK; https://microbesng.com/, accessed on 11 May 2025). The Illumina short-read 30× coverage service or Oxford Nanopore Technologies (ONT) long-read 50× coverage service was used. A total of 7 strain samples were submitted for the Illumina short-read service. Of the 7 samples, parental/untreated *P. aeruginosa* PAO1 and NCTC13437 were included for comparison with the antibiotic-treated isolates. Three of the isolates were from *P. aeruginosa* PAO1, isolated from infected larvae after treatment with ceftazidime, and two came from *P. aeruginosa* NCTC13437, isolated from infected larvae after treatment with meropenem. Three additional isolates were submitted for the ONT long-read service, including parental *P. aeruginosa* PAO1 and two antibiotic-treated isolates from *G. mellonella* larvae following exposure to three doses of ceftazidime over a 96 h period. All strains/isolates were sent in accordance with instructions provided by MicrobesNG. DNA was sequenced by MicrobesNG on an Oxford Nanopore Technologies GridION using R10.4.1 flowcells at a translocation speed of 400 bps. Raw signal was base called using Oxford Nanopore’s basecaller dorado version 0.7.2 (Oxford Nanopore Technologies, Oxford, UK, 2022) and model ‘dna_r10.4.1_e8.2_400bps_sup@v4.3.0’ on an NVIDIA Ampere A100 with 80GB of HBM2 memory. The resulting reads were adapter trimmed using porechop version 0.2.4 [56] and filtered using filtlong version 0.2.1 [57] to retain 120 Mb of reads of at least 1000 bp. Sequencing reads were assembled using flye version 2.9.4 [58], which produced a single circular 6.2-Mb contig. The assembly was polished using medaka version 1.11.3 (Oxford Nanopore Technologies, 2024) and re-circularized to begin at *dnaA* using circlator version 1.5.5 [59]. The final assembly was annotated using bakta version 1.9.4 [60]. Short-read variant calling was performed to identify potential mutations between isolates 6, 9, 12, 14 and 15 and their respective untreated parental strains using snippy version 4.6.0 (https://software.cqls.oregonstate.edu/updates/snippy-4.6.0/, accessed on 11 May 2025; [61]) with default parameters. Long-read variant calling was performed on isolates 27 and 30 against untreated parental *P. aeruginosa* PAO1 using medaka_haploid_variant with model ‘r1041_e82_400bps_sup_variant_v4.3.0’ from medaka version 1.11.3. SnpEff version 5.0 [62] was used to predict the effects of variants on protein-coding genes. To test for structural variants, Oxford Nanopore sequencing reads were aligned to the reference *P. aeruginosa* PAO1 genome using Minimap2 [63] and analysed with Sniffles2 version 2.4 [64].

### 4.9. Analysis of P. aeruginosa MPAO1 with bdkB Knockout

Four strains were purchased from the arrayed *P. aeruginosa* MPAO1 Two-Allele Transposon Mutant Library from Salipante labs (University of Washington, Seattle, WA, USA; https://sites.google.com/uw.edu/salipante-lab, accessed on 11 May 2025). *P. aeruginosa* PW4821 and PW4822 were both engineered to contain a knockout in *bkdB*. *P. aeruginosa* MPAO1, from which all mutant strains were derived, was used as a control. Additionally, the strain LPAO was included because inactivating mutations in *mexT* associated with *P. aeruginosa* PAO1 have been shown to cause slow growth and reduced survival in frozen stocks. *P. aeruginosa* LPAO is a derivative of MPAO1, in which the inactive *mexS* allele has been reverted, restoring this regulatory system to its functional state and thus was a control to verify MPAO1 had not been impacted by mutations in *mexT* (https://sites.google.com/uw.edu/salipante-lab, accessed on 11 May 2025). Upon receipt, all strains were cultured overnight in MHB at 37 °C prior to the addition of glycerol to 15% and storage at −80 °C. To confirm the presence of a transposon insertion in mutant strains, colony PCR was performed using primers 20 and 21 (Supplementary Table S3), designed to flank the transposon insertion site at *bkdB*, and compared to the parent strain MPAO1. In addition, colony PCR was performed using a flanking primer and a transposon-specific primer, LacZ-138 (Supplementary Table S3). Single colonies of each strain isolated on LB agar plates (Thermo Scientific, Loughborough, UK) were resuspended in 10 µL of dH_2_O and 100 µL of lysis buffer (7.0 mM Tris pH 8.0, 3 mM MgCl_2_, 0.5% Tween) was then added, followed by 0.5 µL of 20 mg/mL proteinase K (Thermo Scientific, Loughborough, UK), and incubated at 56 °C for 1 h followed by 15 min incubation at 95 °C. The mixture was then cooled on ice for 5 min, centrifuged at 13,000× *g* for 3 min, and 5 µL of supernatant was used in a 25 µL PCR reaction with 6.1 µL water and 12.5 µL of 2× CloneAmp HiFi PCR premix solution (Takara Bio, San Jose, CA, USA) and 0.7 µL at 10 µM. Thermal cycling was conducted with a 3 min denaturation step at 98 °C, followed by 30 cycles with 10 s at 98 °C, a 15 s annealing step at 50 °C and a 25 s extension step at 72 °C. All PCR products were stored at −20 °C. Successful PCR was determined by gel electrophoresis (1% agarose). Briefly, 5 µL of PCR products were combined with 6× purple loading dye (New England Biolabs, Hitchin, UK) and run on a gel containing SYBR Safe DNA Gel Stain (Thermo Scientific). Gels were subsequently visualised under UV light and product size estimated by comparison to GeneRuler 1kb plus DNA ladder (Thermo Scientific). To determine if the MPAO1 phenotype had been altered due to inactivating mutations in *mexT*, the susceptibility of MPAO1 and LPAO to chloramphenicol and their growth rate in MHB was compared as recommended by the Salipante lab. A sample of 5 mL of MHB was inoculated with each strain and grown overnight at 37 °C. Each strain was diluted down to an OD_600_ of 0.015 and 10 µL plated onto LB (Thermo Scientific) agar containing 10 µg/mL chloramphenicol and incubated overnight at 37 °C. The next day, the plates were inspected for changes to colony count and size compared to LPAO to determine if any displayed reduced growth rate and increased susceptibility to chloramphenicol. The growth rate of MPAO1 and LPAO was determined as described above. The effect of *bkdB* gene knockout on *P. aeruginosa* MPAO1 was determined by measuring the in vitro killing kinetics of ceftazidime against each strain in a time-kill assay, as described above. All experiments were performed in duplicate and the mean and ±SEM calculated using Microsoft Excel.

## Figures and Tables

**Table 1:** The minimum duration to kill 99% (MDK_99_) of *P. aeruginosa* PAO1 and NCTC13437 exposed to MEM or CAZ during time-kill assays. MEM—meropenem; CAZ—ceftazidime.

*P. aeruginosa* Strain	Antibiotic Treatment	MDK_99_ (h) ± SEM
PAO1	MEM (2 mg/L)	4.7 ± 0.3
PAO1	CAZ (4 mg/L)	3.8 ± 0.1
NCTC13437	MEM (128 mg/L)	4.0 ± 0.1
NCTC13437	CAZ (2500 mg/L)	5.4 ± 0.2

**Table 2:** Phenotypic analysis of surviving *P. aeruginosa* PAO1 isolates from infected *G. mellonella* larvae after treatment with three doses of CAZ (3 × 0.25 mg/kg) after 96 h compared with the untreated parent strain. Each isolate was from a single colony recovered from a single healthy larva, subsequently recultured in MHB and stored at −70 °C. Isolates were subsequently recultured in MHB from this freezer stock and growth rate in MHB, time-kill assays in the presence of CAZ (MDK_99_), MIC of CAZ, recovery at 24 h (yes means the log_10_ cfu/mL value in the presence of antibiotic at the 24 h time point in time-kill assays was 2 log_10_ cfu/mL > than the value measured after the 8 h time point) and virulence in *G. mellonella* were measured.

*P. aeruginosa* PAO1 and In Vivo Antibiotic Treatment	Growth Rate (ΔOD_600_/h)	Reduction in Growth Rate (%)	MDK_99_ (h)	Increase in MDK_99_ (%)	Recovery at 24 h (Log_10_ cfu/mL)	Change in MIC	Change in Virulence
Untreated parent control	0.327	0	3.8	0	0	0	0
Isolate 26 CAZ—0.25 mg/kg	0.261	20.2	5.1	34	No (2.0)	No	No
Isolate 27 CAZ—0.25 mg/kg	0.259	20.8	5.3	40	No (2.3)	No	No
Isolate 28 CAZ—0.25 mg/kg	0.249	23.8	5.4	42	No (3.0)	No	No
Isolate 29 CAZ—0.25 mg/kg	0.254	22.3	5.3	40	No (2.8)	No	No
Isolate 30 CAZ—0.25 mg/kg	0.240	26.6	6.0	58	No (3.3)	No	No

**Table 3:** Single nucleotide polymorphisms identified by long-read whole genome sequencing of antibiotic-tolerant isolates 27 and 30 isolated from *G. mellonella* larvae infected with *P. aeruginosa* PAO1 after exposure to three doses of CAZ (3 × 0.25 mg/kg). Variant calling was conducted using medaka version 1.11.3 to identify single polynucleotide polymorphisms, insertions and deletions between next generation sequencing reads compared with the untreated parent strain of *P. aeruginosa* PAO1.

Recovered Isolate	NCBI Locus Tag	Position of Mutation	Type of Mutation	Parental Genotype	Isolate Genotype	% of Isolate Population Carrying Mutation	Gene	Biological Function
Isolate 27	PA2249	3038686	snp	C	T	70	*bdkB*; lipoamide acyltransferase component of branched-chain alpha-keto acid dehydrogenase complex	Catalyses the conversion of alpha keto acids into acyl-CoA and CO_2_
Isolate 30	PA2249	3038686	snp	C	T	51	As above	As above

## Data Availability

The datasets generated during and/or analysed during the current study are available from the corresponding author on reasonable request.

## Supplementary Materials

The following supporting information can be downloaded at https://www.mdpi.com/article/10.3390/antibiotics14050507/s1: Table S1: Phenotypic analysis of surviving *P. aeruginosa* NCTC13437 isolates from infected *G. mellonella* larvae after a single-dose treatment of CAZ (10 mg/kg), or MEM (5 mg/kg) after 96 h, compared with the untreated parent strain; Table S2: Phenotypic analysis of surviving *P. aeruginosa* PAO1 isolates from infected *G. mellonella* larvae after a single-dose treatment of CAZ (0.25 mg/kg) after 96 h, or MEM (0.375 mg/kg) after 24 h, compared with the untreated parent strain; Table S3: List of primers used in this work; Table S4: Phenotypic analysis of *P. aeruginosa* mutant strains containing *bkdB* knockout compared with the parent strain MPAO1.
