# Supplementary material for: Surviving Colonies of Pseudomonas aeruginosa Isolated In Vivo from Infected, Antibiotic-Treated Galleria mellonella Larvae Acquire an Antibiotic-Tolerant Phenotype
Source: Antibiotics (Basel). 2025 May 15;14(5):507. doi: 10.3390/antibiotics14050507 (PMC12108411; doi:10.3390/antibiotics14050507)
Supplement: Supplementary file 1 [file antibiotics-14-00507-s001.zip › antibiotics-3598089-supplementary.pdf]

Supplementary Table S1. Phenotypic analysis of surviving *P. aeruginosa* NCTC13437 isolates from infected *G. mellonella* larvae after a single-dose treatment of CAZ (10 mg/kg) or MEM (5 mg/kg) after 96 h, compared with the untreated parent strain. Each isolate was from a single colony recovered from a single healthy larva that was cultured in MHB and stored at -70°C. Isolates were subsequently recultured in MHB from this freezer stock and growth rate in MHB, time-kill assays in the presence of CAZ or MEM (MDK_{99}), MIC of CAZ and MEM, recovery at 24 h (yes means the log_{10} cfu/mL value in the presence of antibiotic at the 24 h time point in time-kill assays was 2 log_{10} cfu/mL > than the value measured after the 8 h time point), and virulence in *G. mellonella* measured. *Reinfection of *G. mellonella* larvae with isolate 7 showed a 27% increase in survival after 96 h compared with infection with the untreated parent NCTC13437, n=15.

***P. aeruginosa* NCTC13437 and *in vivo* antibiotic treatment**	**Growth rate (ΔOD_{600}/h)**	**Reduction in growth rate (%)**	**MDK_{99} (h)**	**Increase in MDK_{99} (%)**	**Recovery at 24 h (Log_{10} cfu/mL)**	**Change in MIC**	**Change in virulence**
Untreated parent control	0.20	N/A	5.4	N/A	N/A	N/A	N/A
Isolate 1 CAZ – 10 mg/kg	0.12	40	6.7	24.2	Yes (6.5)	No	No
Isolate 2 CAZ – 10 mg/kg	0.125	37.5	5.9	9	Yes (6.5)	No	No
Isolate 3 CAZ – 10 mg/kg	0.103	48.5	5.7	6	Yes (5.8)	No	No
Isolate 4 CAZ – 10 mg/kg	0.171	14.5	7.1	31	Yes (8.4)	No	No
Isolate 5 CAZ – 10 mg/kg	0.184	8	10.8	100	Yes (8.6)	No	No
Untreated parent control	0.2	N/A	4.0	N/A	N/A	N/A	N/A
Isolate 6 MEM – 5 mg/kg	0.185	7.5	9.9	148	Yes (8.0)	No	No
Isolate 7 MEM – 5 mg/kg	0.184	8	6.8	70	No (4.0)	No	*Yes
Isolate 8 MEM - 5 mg/kg	0.192	4	6.6	65	No (4.7)	No	No
Isolate 9 MEM – 5 mg/kg	0.176	12	9.5	137	No (5.7)	No	No
Isolate 10 MEM- 5 mg/kg	0.182	9	6.3	58	No (2.0)	No	No

Supplementary Table S2. Phenotypic analysis of surviving *P. aeruginosa* PAO1 isolates from infected *G. mellonella* larvae after a single-dose treatment of CAZ (0.25 mg/kg) after 96 h, or MEM (0.375 mg/kg) after 24 h, compared with the untreated parent strain. Each isolate was from a single colony recovered from a single healthy larva, subsequently recultured in MHB and stored at -70°C. Isolates were subsequently recultured in MHB from this freezer stock and growth rate in MHB, time-kill assays in the presence of CAZ or MEM (MDK_{99}), MIC of CAZ and MEM, recovery at 24 h (yes means the log_{10} cfu/mL value in the presence of antibiotic at the 24 h time point in time-kill assays was 2 log_{10} cfu/mL > than the value measured after the 8 h time point), and virulence in *G. mellonella* measured.

***P. aeruginosa* PAO1 and *in vivo* antibiotic treatment**	**Growth rate (ΔOD_{600}/h)**	**Reduction in growth rate (%)**	**MDK_{99} (h)**	**Increase in MDK_{99} (%)**	**Recovery at 24 h (Log_{10} cfu/mL)**	**Change in MIC**	**Change in virulence**
Untreated parent control	0.327	N/A	3.8	N/A	N/A	N/A	N/A
Isolate 11 CAZ – 0.25 mg/kg	0.149	54.4	5.6	47	No (3.3)	No	No
Isolate 12 CAZ – 0.25 mg/kg	0.166	49.2	5.9	55	Yes (6.0)	No	No
Isolate 13 CAZ – 0.25 mg/kg	0.178	45.5	5.4	42	No (2.3)	No	No
Isolate 14 CAZ – 0.25 mg/kg	0.153	53.2	5.6	47	No (2.5)	No	No
Isolate 15 CAZ – 0.25 mg/kg	0.174	46.8	6.0	58	No (2.3)	No	No
Untreated parent control	0.327	N/A	4.7	N/A	N/A	N/A	N/A
Isolate 21 MEM – 0.375 mg/kg	0.274	16.2	5.8	21	No (2.0)	No	No
Isolate 22 MEM – 0.375 mg/kg	0.289	11.6	6.9	44	No (3.3)	No	No
Isolate 23 MEM – 0.375 mg/kg	0.292	10.7	6.2	29	No (2.3)	No	No
Isolate 24 MEM – 0.375 mg/kg	0.277	15.3	5.5	15	No (3.0)	No	No
Isolate 25 MEM- 0.375 mg/kg	0.289	11.6	5.3	10	No (2.7)	No	No

Supplementary Table S3. List of primers used in this work. Primers were designed in line with the recommended guidelines provided by the In-Fusion® HD Cloning Kit (Takara Bio; https://www.takarabio.com/).

**Primer No.**	**Primer name**	**Primer sequence 5′ – 3′**
20	*bkdB* fwd (flanking)	GAGCGACACGACGAACAG
21	*bkdB* rev (flanking)	ATCGACCCCGTGTTTCTTC
22	LacZ-138 (transposon specific)	GGGTAACGCCAGGGTTTTCC

Supplementary Table S4. Phenotypic analysis of *P. aeruginosa* mutant strains containing *bkdB* knockout compared to parent strain MPAO1. MKD$_{99}$ values were calculated from percentage survival during time kill assays after exposure to ceftazidime at 4 mg/L and compared with the parent strain. Growth rate was monitored over a 5 h period with measurements taken every hour. Experiments were performed in triplicate and the mean ±SEM is shown.

***P. aeruginosa* strain**	**Growth rate (ΔOD_{600}/h)**	**MDK$_{99}$ (h)**	**Change in MDK$_{99}$ compared with parent strain (%)**
MPAO1	0.151±0.01	5.4±0.5	N/A
PW4821 (*bdkB* insertion knockout)	0.150±0.005	5.6±0.6	3.7
PW4822 (*bdkB* insertion knockout)	0.154±0.001	5.4±0.6	0
